# Targeting Oncoprotein Stability Overcomes Drug Resistance Caused by FLT3 Kinase Domain Mutations

**DOI:** 10.1371/journal.pone.0097116

**Published:** 2014-05-21

**Authors:** Chuanjiang Yu, Rama Krishna Kancha, Justus Duyster

**Affiliations:** Department Medicine I, University Medical Center Freiburg, Freiburg, Germany; Centro de Biología Molecular Severo Ochoa (CSIC-UAM), Spain

## Abstract

FLT3 is the most frequently mutated kinase in acute myeloid leukemia (AML). Internal tandem duplications (ITDs) in the juxta-membrane region constitute the majority of activating FLT3 mutations. Several FLT3 kinase inhibitors were developed and tested in the clinic with significant success. However, recent studies have reported the development of secondary drug resistance in patients treated with FLT3 inhibitors. Since FLT3-ITD is an HSP90 client kinase, we here explored if targeting the stability of drug-resistant FLT3 mutant protein could be a potential therapeutic option. We observed that HSP90 inhibitor treatment resulted in the degradation of inhibitor-resistant FLT3-ITD mutants and selectively induced toxicity in cells expressing FLT3-ITD mutants. Thus, HSP90 inhibitors provide a potential therapeutic choice to overcome secondary drug resistance following TKI treatment in FLT3-ITD positive AML.

## Introduction

Constitutive activation of the FLT3 receptor kinase due to internal tandem duplication (ITD) or point mutation (D835Y) is detected in almost 30% of AML patients [1]. Hereby, FLT3-ITD is the most frequent genetic alteration and was found to be associated with a poor prognosis thus making it a potential therapeutic target [1], [2]. Inhibitors that target the FLT3 kinase activity have been developed and tested within clinical trials with significant success[3]–[5]. However, responses seen with FLT3 inhibitors were only transient. Studies using *in vitro* cell-based screening techniques have predicted FLT3-ITD kinase domain mutations that cause secondary drug resistance [6], [7]. In line with these *in vitro* studies, emergence of secondary drug resistant mutations were reported in patients treated with FLT3 inhibitors[8]–[11]. Novel inhibitors are able to overcome drug resistance caused by secondary FLT3-ITD kinase mutations in some cases [12], [13]. However, many kinase domain mutations exhibit inhibitor cross-resistance[7], [10], [12], [14]–[16]. Thus, there is a need to search for alternate means to overcome secondary drug resistance caused by FLT3 kinase domain mutations.

It was previously shown that FLT3-ITD is a client kinase for the HSP90 chaperone [17]. Subsequent studies have shown that the HSP90-FLT3-ITD interaction is sensitive to HSP90 inhibitors resulting in selective toxicity towards FLT3-ITD positive cells [17], [18]. Earlier studies have shown that the HSP90-kinase interaction is mediated by the kinase domain [19]. We thus tested if inhibitor-resistant FLT3 kinase domain mutants are stabilized by HSP90.

## Materials and Methods

### DNA Constructs, Cell Lines and Chemical Reagents

MiGR1-FLT3-D835Y and MiGR1-FLT3-ITD constructs were described previously [7], [12]. FLT3-ITD-N676K was created using QuickChangeSite-Directed Mutagenesis Kit (Stratagene, Germany) according to manufacturer’s instructions [12].

32D cells were cultured in RPMI-1640 medium (Life Technologies) supplemented with 10% FCS and glutamine. Parental 32D cells were cultured in interleukin-3 (IL-3, R&D Systems). 32D cells stably expressing FLT3 mutants were established by retroviral infection followed by IL-3 withdrawal [12].

Geldanamycin and 17-AAG (Tanespimycin) were purchased from InvivoGen, USA. 17-DMAG (Alvespimycin) was purchased from Biozol Diagnostica Vertrieb GmbH, Germany. All HSP90 inhibitors were dissolved in DMSO (at 1 mmol/L for geldanamycin and 17-AAG and at 10 mmol/L for 17-DMAG) and stored at −20°C.

### Immunoprecipitation and Western Blotting

MiGR1-FLT3 DNA constructs were transfected into HEK293 cells with Lipofectamine 2000 reagent (Invitrogen) for 36 hours followed by cell lysis with TMNSV buffer (50 mM Tris-HCl pH-7.5, 20 mM Na_2_MoO_4_, 0.09% Nonidet P-40, 150 mM NaCl and 1 mM Sodium orthovanadate). Cells were then immunoprecipitated with goat anti-FLT3 antibody. SDS-PAGE and western blotting were performed as described before [12].

For protein degradation analysis, 32D cells expressing FLT3 mutants were treated with indicated HSP90 inhibitors for 12 hours followed by cell lysis in buffer containing 10 mM Tris-HCl pH-7.5, 130 mM NaCl, 5 mM EDTA, 0.5% Triton X-100, 20 mM Na_2_HPO_4_/NaH_2_PO_4_ pH-7.5, 10 mM sodiumpyrophosphate pH-7.0, 1 mM Sodiumorthovanadate, 20 mM Sodium fluoride and 1 mM Glycerol-2-phosphate.

Following antibodies were used for immunoblotting: mouse anti-FLT3 (Upstate Biotechnology), mouse anti-HSP90 (F-8 from Santa-Cruz biotechnology), mouse anti-Cdc37 (E-4 from Santa-Cruz biotechnology), rabbit anti-pSTAT5-Tyr694 (Cell Signaling), rabbit anti-STAT5 (Santa Cruz Biotechnology), rabbit anti-pERK1/ERK2 (Cell Signaling), and rabbit anti-ERK1/ERK2 (Cell Signaling). Bands were visualized using the enhanced chemiluminiscence system (Amersham).

### Cell Death Assay and Drug Resistance Assay

32D cells stably expressing FLT3 mutants were treated with indicated concentrations of HSP90 inhibitors for 48 hours and cell death was measured by propidium-iodide (Sigma) staining and FACS analysis [12].

To test for the emergence of drug resistance, a cell-based screen was performed as described previously [7]. Briefly, 4×10^5^ cells per well were cultured in the presence of 50 nM sorafenib either alone or in combination with an HSP90 inhibitor (250 nM of geldanamycin or 2000 nM of 17-AAG). Development of drug-resistant colonies was analyzed after 3 weeks of culture.

## Results and Discussion

**Figure 1:**
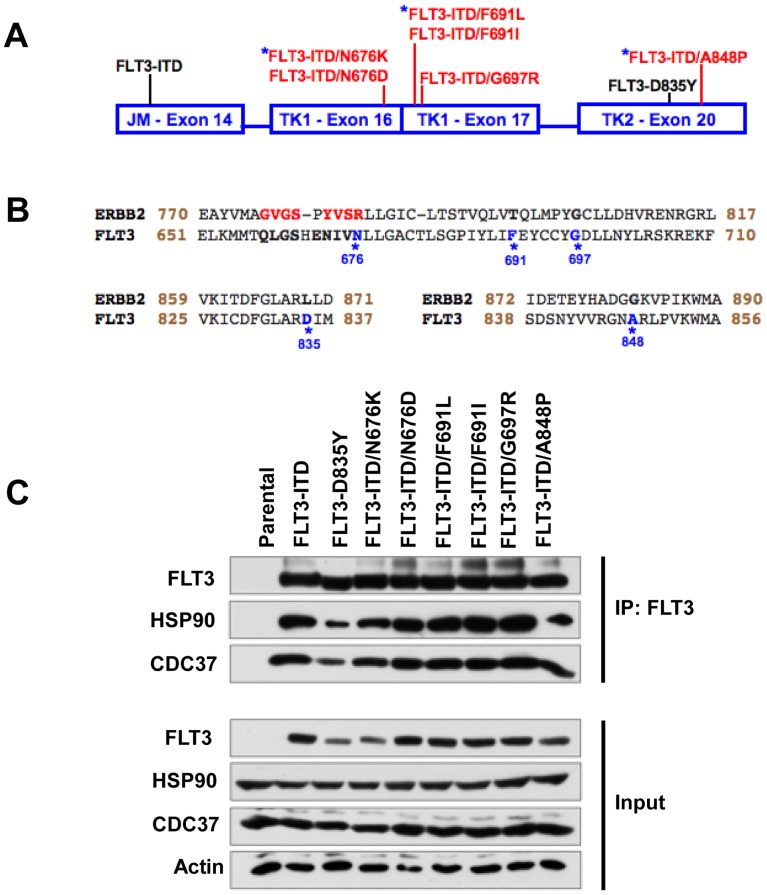
Kinase inhibitor resistant FLT3-ITD mutants retain interaction with HSP90. (A) Panel of FLT3 mutations investigated in the study were indicated. Kinase inhibitor sensitive FLT3 mutants were shown in black while drug resistant FLT3 mutants were shown in red. Kinase domain mutations that were shown to cause secondary drug resistance were marked with a blue asterisk. (B) Comparison of the amino acid sequence of FLT3 kinase domain to that of the ERBB2 kinase domain. Amino acid residues that were important for HSP90 interaction were highlighted in red and mutations at the amino acids that cause drug resistance were indicated in blue. (C) HEK293 cells were transfected with FLT3 mutants and immunoblotting on FLT3 immunoprecipitates was performed using indicated antibodies.

The aim of this study was to examine the interaction between HSP90 and secondary FLT3-ITD mutants that confer resistance to FLT3 kinase inhibitors. Several drug-resistant FLT3 mutants were reported both in patients and in *in vitro* drug resistance screens[6], [8]–[11], [14], [20]. The position of the secondary FLT3 mutations conferring TKI resistance examined in this study are schematically represented in Figure 1A in red [6]–[12], [14], [20]. The position of the activating FLT3-ITD and FLT3-D835Y mutation are indicated in black. Inhibitor-resistant FLT3 mutations that were reported in AML patients are marked by a blue asterisk (Figure 1A) [8], [9], [11], [20]. FLT3-N676K was reported to cause secondary resistance in an AML patient who was treated with PKC412 [8]. FLT3-ITD-A848P was reported in a chronic myelomonocytic leukemia (CMML) patient at blast phase and was shown to cause resistance towards sunitinib and sorafenib [20]. FLT3-F691L is a gate-keeper mutation that was reported in several AML patients who relapsed after treatment with the FLT3 inhibitor AC220 [9], [11].

All drug-resistant mutations are located in the kinase domain (TK1 or TK2 of the split-kinase domain) (Figure 1A). It has been previously shown for ERBB2 that the kinase domain mediates the client interaction with the HSP90 chaperone [19]. The amino acids in the ERBB2 kinase domain critical for binding to HSP90 are marked red in Fig. 1B. FLT3-N676 is in the region that was reported to be critical for ERBB2 binding to HSP90 chaperone (Figure 1B). We first tested if the kinase inhibitor resistant FLT3-ITD mutants interact with HSP90. FLT3 mutants were transfected into HEK293 cells and FLT3 immunoprecipitates showed that all the mutants tested interacted with HSP90 and the co-chaperone CDC37 (Figure 1C). This indicates that drug-resistant FLT3 mutants still depend on HSP90 for their stability.

**Figure 2:**
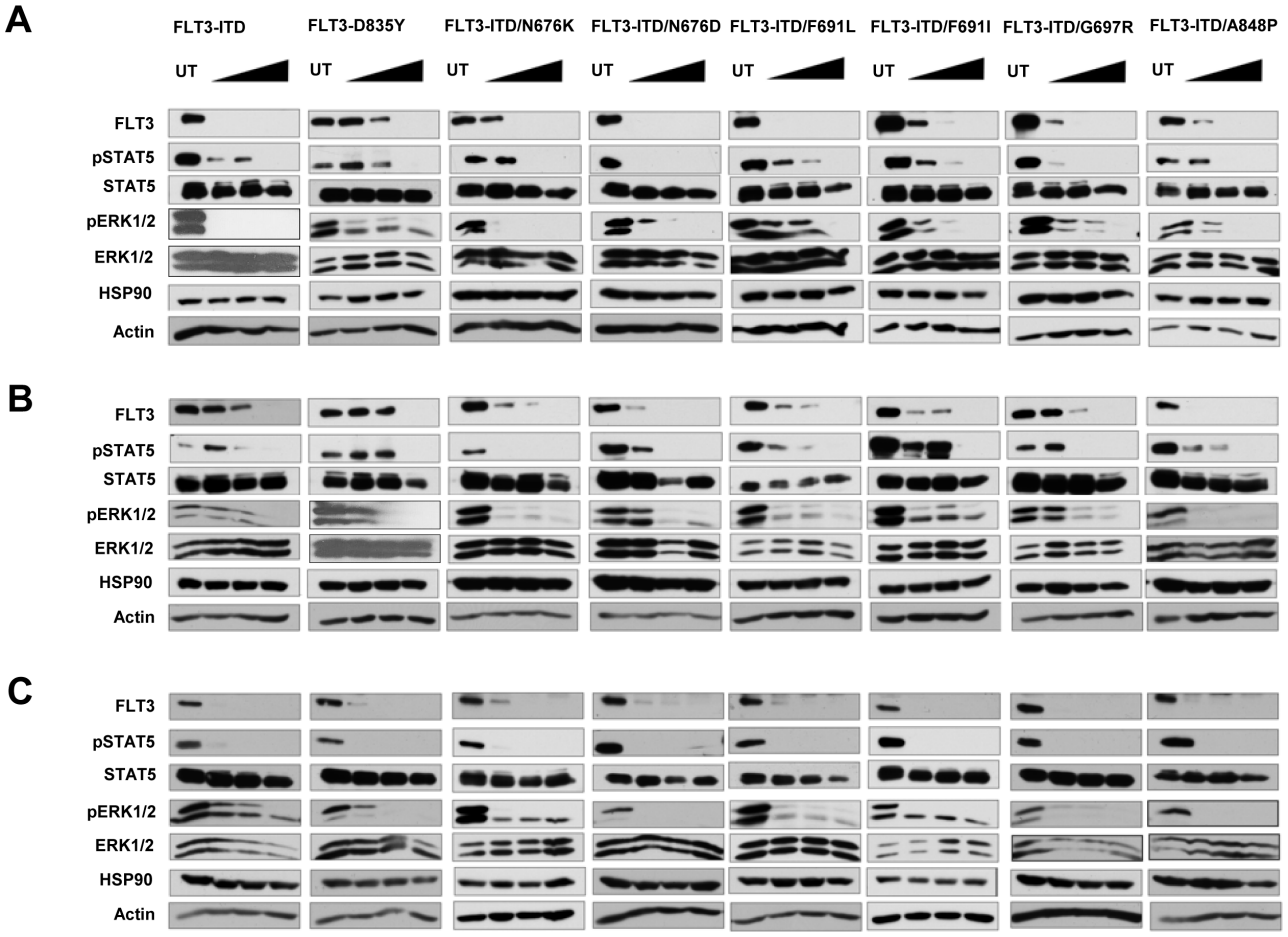
HSP90 inhibitor treatment causes degradation of FLT3 mutants. 32D cells stably expressing FLT3 mutants were treated with increased concentrations (250 nM, 500 nM and 1000 nM) of HSP90 inhibitors Geldanamycin (A), 17-AAG (B) or 17-DMAG (C) for 12 h and analyzed by western blotting with indicated antibodies.

It was previously shown that HSP90 inhibitor treatment results in the degradation of FLT3-ITD [17]. Treatment of 32D cells stably expressing various FLT3 mutants with increasing concentrations of HSP90 inhibitors geldanamycin (Figure 2A), 17-AAG (Figure 2B) or 17-DMAG (Figure 2C) resulted in the degradation of the kinase. The activation of downstream signaling molecules STAT5 and ERK1/2 was inhibited in a dose-dependent manner in all cell lines tested (Figure 2A–C).

**Figure 3:**
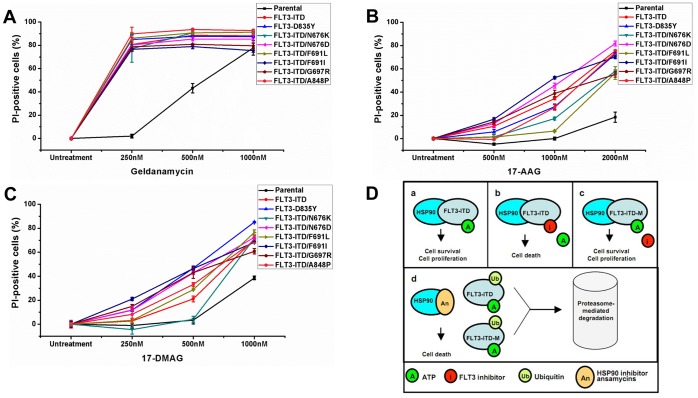
HSP90 inhibitors induce selective toxicity in cells expressing FLT3 mutants. 32D cells stably expressing FLT3 mutants were treated with HSP90 inhibitors Geldanamycin (A), 17-AAG (B) or 17-DMAG (C) for 48 hours and analyzed by FACS following propidium iodide staining. (D) Schematic representation of the efficacy of HSP90 inhibitors against both the kinase inhibitor sensitive as well as inhibitor resistant FLT3 mutants: (a) FLT3-ITD is stabilized by HSP90 resulting in cellular transformation, (b) FLT3-ITD is sensitive to kinase inhibitors that lead to death of cells expressing them, (c) Secondary mutations in FLT3-ITD kinase domain abrogate inhibitor binding leading to resistance towards kinase inhibitors and (d) Treatment with HSP90 inhibitors leads to degradation of both FLT3-ITD as well as drug-resistant mutants resulting in selective cytotoxicity towards FLT3-expressing cells.

To test the selective potency of HSP90 inhibitors to induce apoptosis in FLT3-ITD expressing cells, we treated 32D cells stably expressing TKI resistant FLT3-ITD mutants. HSP90 inhibitors (geldanamycin, 17-AAG and 17-DMAG) induced significant cell death in both the drug-sensitive and drug-resistant FLT3 mutants (Figure 3A–C). 32D parental cells were less sensitive to HSP90 inhibitor treatment than FLT3 overexpressing cells (Figure 3A–C). Geldanamycin was the most potent HSP90 inhibitor tested with selective cytotoxicity against FLT3 expressing cells observed at concentrations as low as 250 nM (Figure 3A). There was no significant difference between FLT3-ITD (red), FLT3-D835Y (blue) and drug resistant FLT3-ITD mutants regarding geldanamycin sensitivity (Figure 3A and Table 1). While FLT3-ITD (red) and FLT3-D835Y (blue) displayed very high sensitivity towards 17-AAG treatment, the efficacy was slightly lower against FLT3-ITD-N676K and FLT3-ITD-F691L at 1 µM (Figure 3B). However, significant selective toxicity of 17-AAG towards FLT3 expressing 32D cells was observed at 2 µM (Figure 3B and Table 1). Similarly, 17-DMAG was highly potent against all the FLT3 expressing 32D cell lines at 1 µM but the selectivity with respect to parental 32D cells is lower when compared to the HSP90 inhibitors geldanamycin and 17-AAG (Figure 3C and Table 1). Thus, we propose that HSP90 inhibitors may be used to target FLT3 mutants irrespective of their sensitivity towards FLT3 inhibitors (Figure 3D).

**Table 1:** HSP90 inhibitor sensitivity profiles for FLT3 mutations.

FLT3 mutation	Geldanamycin (0.25 µM)	Tanespimycin (2 µM)	Alvespimycin (1 µM)
**ITD**	78.7	57.1	32.2
**D835Y**	82.7	54.2	46.5
**ITD/N676K**	75.0	39.7	32.9
**ITD/N676D**	78.3	63.2	33.9
**ITD/F691L**	84.1	37.9	37.9
**ITD/F691I**	74.6	51.8	21.9**
**ITD/G697R**	76.7	36.8	22.2**
**ITD/A848P**	87.8	55.6	36.7

Selective effect of HSP90 inhibitors was calculated as a difference in cell death between parental 32D cells and 32D-FLT3 mutant cells. Increased levels in cell death of 32D-FLT3 mutant cells when compared to parental 32D cells were shown.

**FLT3-ITD-F691I and FLT3-ITD-G697R were more sensitive to alvespimycin treatment at 0.5 µM concentration (42.9% and 39.5% increase for F691I and G697R respectively).

It has been proposed that the “chaperone addiction” observed in cancer cells can be exploited for selective therapeutic targeting [21]. Previous studies have reported the efficacy of HSP90 inhibitors towards mutant FLT3 either alone or in combination with FLT3 inhibitors [17], [18], [22], [23]. Moreover, several HSP90 inhibitors are currently investigated within clinical trials [24]. This raises the possibility of using HSP90 inhibitors to overcome secondary drug resistance against FLT3 inhibitors that arise due to kinase domain mutations. In this study we show that targeting oncoprotein stability using HSP90 inhibitors overcomes drug resistance caused by FLT3 kinase domain mutations. In addition, we performed a drug-resistance assay to test if the combination of a FLT3 kinase inhibitor (sorafenib) and an HSP90 inhibitor (geldanamycin or 17-AAG) prevents the emergence of secondary drug resistance. As described before, a significant number of sorafenib-resistant clones were observed when Ba/F3-FLT3-ITD cells were cultured at 50 nM (Figure S1). Importantly, no resistant clones emerged when Ba/F3-FLT3-ITD cells were cultured in the presence of a combination of 50 nM of sorafenib and either 250 nM of geldanamycin (Figure S1) or 2000 nM of 17-AAG (Figure S1). Thus, simultaneous targeting of multiple properties (for example enzyme activity as well as the stability of a protein) of an oncoprotein may prevent the emergence of secondary drug resistance in AML patients.

## Supporting Information

Figure S1 **A combination of FLT3 kinase inhibitor and HSP90 inhibitor prevents the emergence of secondary drug resistance.** 4×10^5^Ba/F3-FLT3-ITD cells were plated and cultured in 50 nM of sorafenib (A) either alone or in combination with an HSP90 inhibitor (250 nM of geldanamycin (B) or 2000 nM of 17-AAG (C)) for three weeks. MTS substrate was then added to cells and drug-resistant clones were analyzed.
(TIF)
