# Supplementary figures and images for: Targeting Oncoprotein Stability Overcomes Drug Resistance Caused by FLT3 Kinase Domain Mutations
Source: PLoS One. 2014 May 21;9(5):e97116. doi: 10.1371/journal.pone.0097116 (PMC4029991; doi:10.1371/journal.pone.0097116)

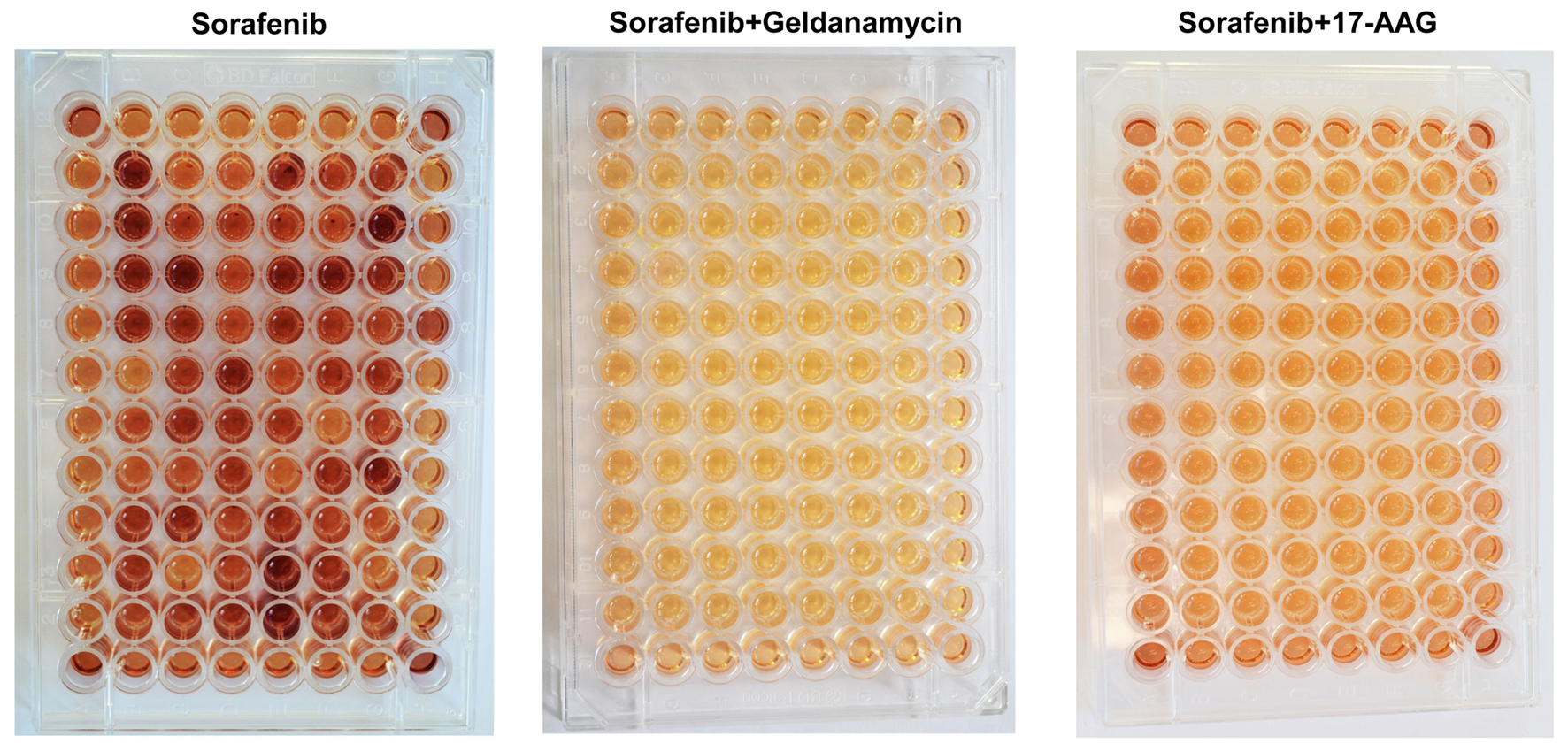

Supplement: Figure S1 — A combination of FLT3 kinase inhibitor and HSP90 inhibitor prevents the emergence of secondary drug resistance. 4×10^5^Ba/F3-FLT3-ITD cells were plated and cultured in 50 nM of sorafenib (A) either alone or in combination with an HSP90 inhibitor (250 nM of geldanamycin (B) or 2000 nM of 17-AAG (C)) for three weeks. MTS substrate was then added to cells and drug-resistant clones were analyzed. (TIF) [file pone.0097116.s001.tif]
